# Optimization of ^99m^Tc-SPECT in the presence of ^90^Y for radioembolization

**DOI:** 10.1186/s40658-025-00798-5

**Published:** 2025-09-25

**Authors:** Camiel E.M. Kerckhaert, Martijn M.A. Dietze, Rob van Rooij, Marjolein B.M. Meddens, Niek Wijnen, Maarten L.J. Smits, Marnix G.E.H. Lam, Hugo W.A.M. de Jong

**Affiliations:** Radiology and Nuclear Medicine, Utrecht University and University Medical Center Utrecht, P.O. Box 85500, Utrecht, 3508 GA Netherlands

**Keywords:** Dual-isotope SPECT, Technetium-99m, Yttrium-90, Radioembolization

## Abstract

**Background:**

^99m^Tc-macroaggregated albumin (MAA) imaging is part of the standard work-up procedure for radioembolization using ^90^Y microspheres. In certain scenarios, it may be warranted to visualize the distribution of ^99m^Tc in co-presence of ^90^Y, for example when validating intra-procedural ^99m^Tc-MAA imaging after ^90^Y-therapy to enable single-session radioembolization. Another instance involves additional ^99m^Tc-MAA administration during the therapeutic procedure itself, e.g. when initial imaging reveals insufficient targeting of a specific liver segment. In these situations, crosstalk from ^90^Y can result in reduced ^99m^Tc image quality and quantitative accuracy. This study investigates the feasibility and optimal method of ^99m^Tc SPECT imaging from combined ^99m^Tc+^90^Y data using phantom experiments.

**Results:**

An anthropomorphic torso phantom with two liver tumor inserts was filled with ^99m^Tc without (single-isotope) and with ^90^Y (dual-isotope) in various activities and isotope concentrations. Three collimators (low energy high resolution: LEHR, medium energy: ME, and high energy: HE) and three methods to compensate for ^90^Y crosstalk in the ^99m^Tc photo peak window (Monte Carlo-based, dual-energy-window and triple-energy-window correction) were evaluated. No substantial dead-time effects were observed in the clinically relevant activity range, up to approximately 12 GBq ^99m^Tc+^90^Y (ratio 1:20) with LEHR, 29 GBq with ME and > 30 GBq with HE. Compared to the clinical standard (single-isotope ^99m^Tc imaging with LEHR collimator), contrast recovery typically decreased from 70.0 ± 1.3% to 49.0 ± 0.9% (LEHR), 61.2 ± 1.5% (ME) or 62.1 ± 1.4% (HE) due to ^90^Y crosstalk. Compensation methods increased contrast recovery, with Monte Carlo-based correction combined with a ME or HE collimator yielding the best recovery at 68.5 ± 1.6% and 68.3 ± 1.5%, respectively. Visual image quality in terms of resolution and scatter contamination was superior when using a ME collimator. Lung shunt fractions were also severely affected by ^90^Y crosstalk when using LEHR, but could be effectively mitigated using a ME or HE collimator.

**Conclusion:**

^99m^Tc imaging in the presence of ^90^Y leads to substantial image degradation due to crosstalk effects. Monte Carlo-based crosstalk compensation in combination with a ME or HE collimator was identified as the most accurate, robust and visually optimal reconstruction method for ^99m^Tc SPECT from dual-isotope data.

**Supplementary Information:**

The online version contains supplementary material available at 10.1186/s40658-025-00798-5.

## Introduction

Radioembolization, or selective internal radiation therapy (SIRT), is a treatment for tumors in the liver using intra-arterially injected yttrium-90 (^90^Y) loaded microspheres [1]. Prior to therapy, patients undergo a work-up procedure in which technetium-99m (^99m^Tc) labeled macro-aggregated albumin (MAA) particles are injected, followed by SPECT/CT. The goals of the work-up procedure are to assess intra-hepatic activity distribution, rule-out potential non-target embolization and establish a treatment plan. In a second procedure, typically 1–2 weeks after the work-up procedure, patients undergo treatment with intra-arterial ^90^Y microsphere injection. Combining the work-up and therapeutic procedure in a single session may hold significant benefits for the patient and healthcare system (e.g. reduction in hospital visits, invasive procedures, costs, time needed in the angiography suite). In our center, efforts are underway to combine ^99m^Tc-MAA and ^90^Y-microsphere injection in a single session using an interventional hybrid C-arm capable of ^99m^Tc-SPECT/CBCT imaging in between injections, enabling real-time treatment planning and immediate therapy delivery [2–4]. To evaluate performance of the hybrid C-arm in a clinical trial, the interventional ^99m^Tc-SPECT/CBCT will be compared to traditional diagnostic ^99m^Tc-SPECT/CT following the combined intervention. This, however, introduces a dual-isotope situation as both ^90^Y and ^99m^Tc are present in the post-procedural SPECT/CT.

While this study focuses on the situation described above, there are similar scenarios in which ^99m^Tc-MAA and ^90^Y-microspheres are intentionally administered within the same procedure. Although such cases are occasional, they underscore the clinical relevance of combined ^99m^Tc/^90^Y imaging. For instance, a second MAA-scout procedure may be performed during the same session as the initial ^90^Y therapy to support sequential super-selective therapies, such as segmentectomy involving multiple liver segments [5]. Another example arises when an initial MAA-scout identified insufficient targeting of certain liver regions, leading to a repeated scout performed in the same session as the treatment of regions that were already deemed suitable [6]. In these cases, combining the second MAA-scout with the first ^90^Y treatment helps to reduce overall procedure time and radiation exposure for the patient, while still requiring accurate ^99m^Tc-SPECT imaging despite the presence of ^90^Y.

^99m^Tc is a widely used radionuclide in diagnostic imaging due to its 6-hour half-life and 140 keV gamma-ray emission, making it ideal for SPECT imaging [7]. In contrast, ^90^Y is a beta-emitting isotope with a 2.66 day half-life and an average beta energy of 0.94 MeV, which is effective in delivering therapeutic radiation [8, 9]. The broad bremsstrahlung spectrum of ^90^Y overlaps with the ^99m^Tc photo peak causing substantial crosstalk when imaging ^99m^Tc. Crosstalk can lead to reduced contrast, quantification errors, degraded spatial resolution and increased background noise in the image reconstruction.

Several studies have improved dual-isotope SPECT by addressing crosstalk for a number of radionuclide combinations. In some cases, optimizing energy window selection is sufficient, e.g. using a high-energy window to reduce ^99m^Tc down scatter in ^99m^Tc/^90^Y dual-isotope imaging [10]. However, unlike the present study, that work focused on improving ^90^Y image quality from ^99m^Tc/^90^Y acquisitions. When spectral overlap is more pronounced, additional energy windows can be used to estimate crosstalk, e.g. in simultaneous ^99m^Tc/^201^Tl, ^99m^Tc/^123^I or ^99m^Tc/^111^In SPECT [11–14]. More advanced approaches, including Monte Carlo-based corrections, have been implemented to further enhance quantification accuracy in dual-isotope imaging, e.g. in simultaneous ^99m^Tc/^201^Tl or ^166^Ho/^99m^Tc SPECT [15, 16]. Our study investigates the feasibility and optimal approach for post-therapy ^99m^Tc SPECT imaging in the co-presence of ^90^Y using phantom experiments.

## Methods

### Phantom experiments

**Fig. 1:**
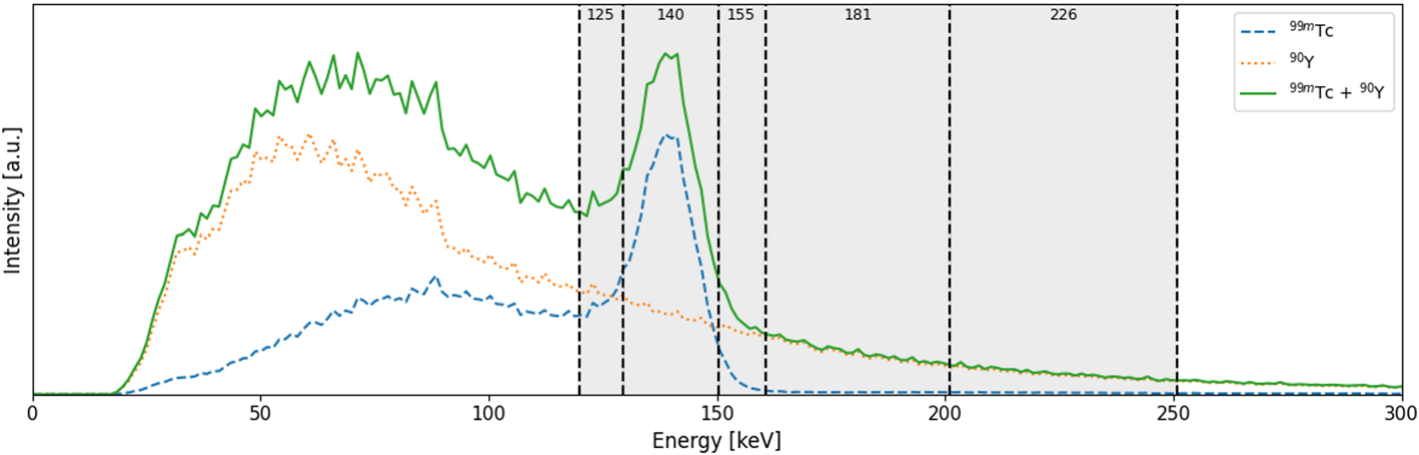
Measured energy spectra of ^99m^Tc (blue, dashed), ^90^Y (orange, dotted), and combined ^99m^Tc+^90^Y (green, solid) in activity ratio 1:20 (0.97 MBq ^99m^Tc, 19.30 MBq ^90^Y). Five energy windows from the spectrum were used in this study, including 125 keV, 140 keV, 155 keV, 181 keV and 226 keV. Spectra were acquired using a Siemens Intevo Bold system without collimator

Data were acquired using a Siemens Intevo Bold SPECT/CT system (Siemens Healthineers, Erlangen, Germany) [17]. Since ^99m^Tc and ^90^Y imaging are performed using different collimators in clinical practice, low-energy high resolution (LEHR; 24.04 mm hole length, 0.16 mm septal thickness, 1.11 mm hole diameter), medium energy (ME; 40.64 mm hole length, 1.14 mm septal thickness, 2.94 mm hole diameter) as well as high energy (HE; 59.7 mm hole length, 2.0 mm septal thickness, 4.0 mm hole diameter) collimators were included. Furthermore, we captured projection data in the full energy window (0-690 keV) and in five smaller windows. These included the 140 keV window (129.5-150.5) to capture the ^99m^Tc photo peak with ^90^Y crosstalk; the 125 keV (119.7-129.5) and 155 keV (150.5-160.3) windows to measure ^99m^Tc scatter and ^90^Y crosstalk; and the 181 keV (161.1-200.9) and 226 keV (201.1-250.9) windows to capture exclusively ^90^Y bremsstrahlung, as shown in Fig. 1 [7, 8, 10]. Taking scan duration and the relatively short ^99m^Tc half-life into account, three scans per collimator were acquired in 120 projections of 20 s in continuous rotation mode.

**Fig. 2:**
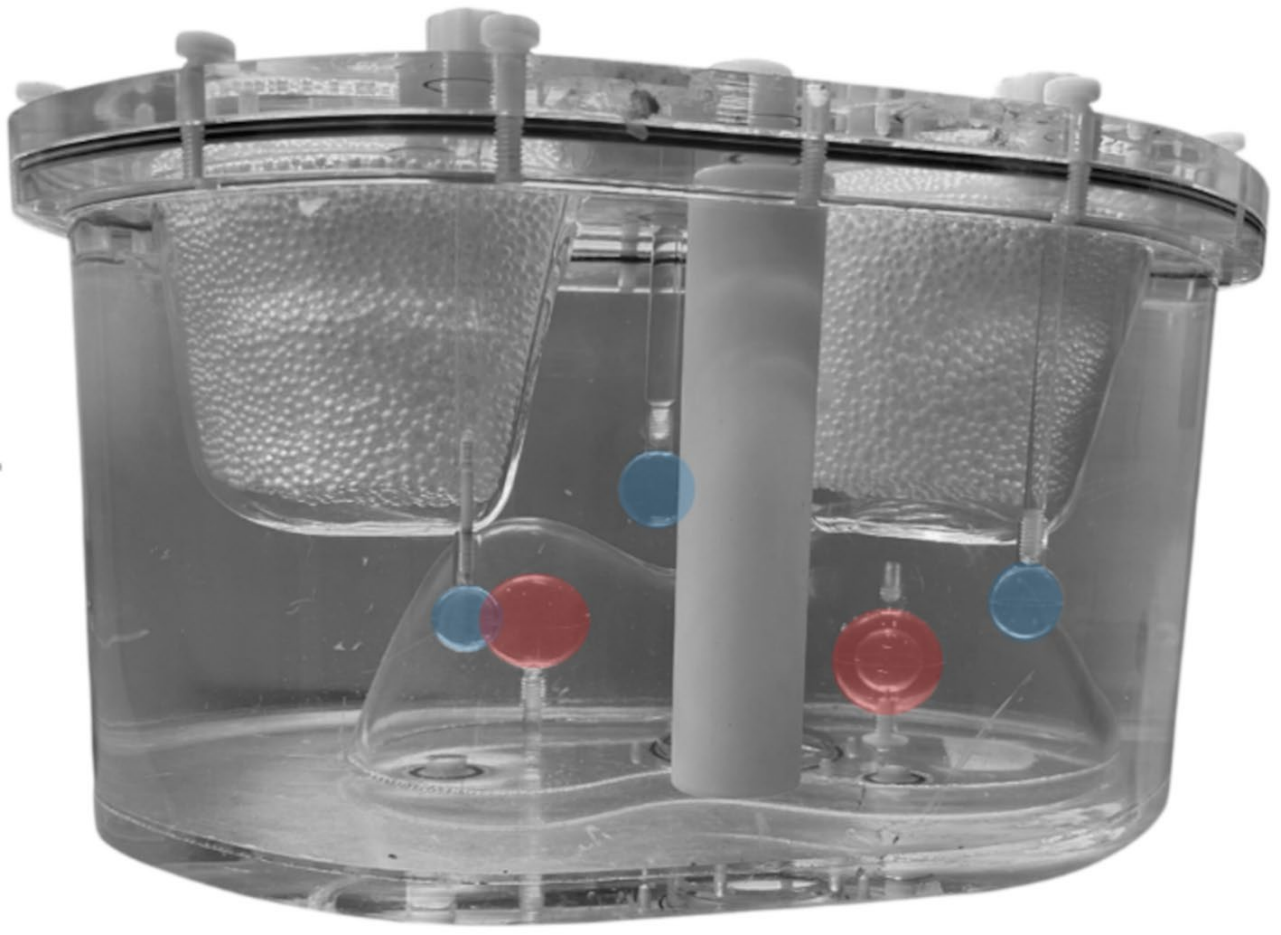
Anthropomorphic torso phantom with liver and lung compartments, liver tumors 1 (right) and 2 (left) in red, and extrahepatic depositions 1 (middle), 2 (right) and 3 (left) in blue

Experiments were performed using an anthropomorphic torso phantom (model ECT/TOR/P, Data Spectrum Corp.) depicted in Fig. 2. The phantom contained a liver insert (1172 mL) with two spheres (24.5 and 16.0 mL) representing liver tumors, with the largest sphere comprising two layers treated as a single sphere. Furthermore, two lung inserts (690 mL in total) and three spheres (8.1, 4.1 and 2.0 mL) in the background volume representing extrahepatic depositions (EHD) were present. Tumor-to-nontumor ratios (T/N ratios), EHD-to-liver activity ratios and lung shunt fractions (LSF) selected to reflect clinically relevant conditions and to assess reconstruction performance under varying isotope distributions are summarized in Table 1. Different LSFs and T/N ratios for ^99m^Tc and ^90^Y enabled evaluation of crosstalk correction accuracy. The three extrahepatic depositions included scenarios with both isotopes, only ^90^Y, or only ^99m^Tc, allowing assessment of the ability to correctly attribute or suppress signal. Phantom experiments using standard ^99m^Tc+^90^Y activity ratios of 1:20 standard were performed, corresponding to a clinically relevant radioembolization dose distribution of 150 MBq ^99m^Tc and 3 GBq ^90^Y in our hospital. The phantom was filled with ^99m^Tc-pertechnetate and ^90^Y-chlorid mixed with hydrochloric acid to prevent adhesion to the phantom walls.

**Table 1:** Phantom configurations for ^99m^Tc and ^90^Y applied across all experiments, including ^99m^Tc-only, ^90^Y-only and simultaneous ^99m^Tc/^90^Y imaging. These configurations were used consistently in the synthetic data validation, the main Phantom study and the experiments with varied ^90^Y:^99m^Tc activity ratios

	^99m^Tc	^90^Y
Tumor 1/Normal liver ratio [-]	4.0	1.0
Tumor 2/Normal liver ratio [-]	4.0	4.0
Lung shunt fraction [%]	15.0	10.0
Extrahepatic deposition 1/liver ratio [%]	3.28	3.13
Extrahepatic deposition 2/liver ratio [%]	0.00	1.58
Extrahepatic deposition 3/liver ratio [%]	0.81	0.00

In addition to dual-isotope filling of the phantom, ^99m^Tc and ^90^Y single-isotope acquisitions were conducted with the same phantom configurations (see Table 1). By combining the two single-isotope data sets we created a synthetic dual-isotope data set that allowed for more flexibility in activity levels and isotope ratios. This ensured a fair comparison between collimators (at equal activity levels), a direct comparison to ^99m^Tc-only reconstructions, and allowed additional experiments to optimize models and assess robustness.

This synthetic approach was based on the assumption that the count rate was linear in all studied situations, meaning no relevant background signal and no dead time effects could be observed. This linearity was tested using intrinsic and extrinsic (for each collimator) planar acquisitions of sixty seconds of ^99m^Tc, ^90^Y and ^99m^Tc+^90^Y point sources in a water-filled cylindrical phantom at sixteen activity levels (0.10-17.15 MBq ^99m^Tc and 2.00-343.00 MBq ^90^Y, maintaining a ^99m^Tc+^90^Y ratio of 1:20). Observed and ideal intrinsic count rates were used to construct intrinsic count rate curves, where ideal count rates were extrapolated from low-activity measurements (< 1 MBq ^99m^Tc and < 20 MBq ^90^Y) assuming negligible dead-time. The intrinsic count rate curves in combination with extrinsic-intrinsic sensitivity ratios were then used to estimate dead-time up to 30 GBq, similarly to a study by Elschot et al. [18].

For validation of this synthetic approach, synthetic dual-isotope reconstructions were directly compared to original dual-isotope image reconstructions. To precisely align activity levels between the measured dual-isotope data and single isotope data used to generate synthetic dual-isotope data, lower activities were emulated by conversion of the projection data to pseudo list-mode data. To this end, all individual counts were put in a list, and a specific fraction was used to form projections with fewer counts effectively representing lower activity. Three measurements per collimator were included, with activities ranging from 69 to 118 MBq for ^99m^Tc and 1922–2048 MBq for ^90^Y.

For the phantom study, the final synthetic dual-isotope dataset was created by summing all acquired single-isotope data per collimator and redistributing in three noise realizations (i.e. separate instances of the same activity distribution with different Poisson noise) per isotope ratio before combining ^99m^Tc and ^90^Y data. Each noise realization included activities of 98 MBq ^99m^Tc and 1969, 3939 or 5909 MBq ^90^Y, corresponding to ratios of 1:20, 1:40 and 1:60, respectively.

### Image reconstruction - ^90^Y Estimation and removal

SPECT images were reconstructed with ordered-subset expectation maximization (OSEM) using the Utrecht Monte Carlo System (UMCS), which includes scatter correction by fast Monte Carlo simulations, attenuation correction and point spread function (PSF) modeling [19, 20]. For each energy window and collimator, PSF kernels including collimator-detector response were pre-calculated using MCNP [21]. Images of 128 × 128 × 128 voxels of 4.8 × 4.8 × 4.8 mm^3^ were reconstructed in 50 iterations of 8 subsets. As the convergence differs per collimator and correction method, images were compared at the iteration that yielded a noise level most closely matching that of the ^99m^Tc-only LEHR reconstruction at ten iterations.

To evaluate the feasibility of ^99m^Tc SPECT imaging in the presence of ^90^Y, five reconstructions were included: (i) reconstruction from ^99m^Tc-only data, (ii) reconstruction from dual-isotope data without ^90^Y crosstalk correction, and three different ^90^Y crosstalk corrections, including (iii) Monte Carlo based correction, (iv) dual energy-window correction, and v) triple energy-window correction. Each correction method predicts the ^90^Y in the 140 keV window, and subsequently removes that from the ^99m^Tc image by iteratively adding it as a background term during reconstruction.

#### Monte Carlo based correction (i.e. MC)

This methods utilized the 226 keV window to predict the ^90^Y in the 140 keV window. The 226 keV window was selected over the 181 keV window because of its lower likelihood of containing ^99m^Tc signal, and could therefore be used to reconstruct a ^90^Y image using Monte Carlo based reconstruction [22]. The iterative ^90^Y reconstruction was halted near convergence of contrast-noise ratio, specifically when the increase was < 2% across three consecutive iterations. From this image, noise-free 140 keV ^90^Y projections were generated using Monte Carlo simulations in UMCS.

#### Dual energy-window correction (i.e. DEW)

This method estimated ^90^Y in the 140 keV window by scaling the 226 keV window using a k-factor [15]. The k-factors were determined from point source measurements of ^90^Y in a cylindrical water-filled phantom, calculated by dividing the total counts in the 140 keV window by those in the 226 keV window. The resulting k-factors were 0.478, 0.632 and 0.745 for the LEHR, ME and HE collimators, respectively.

#### Triple energy-window correction (i.e. TEW)

In this method, the lower and upper scatter windows of ^99m^Tc (125 and 155 keV windows) were used to estimate non primary-^99m^Tc photons in the 140 keV window by averaging the two windows. Since this estimation included both ^90^Y and scattered ^99m^Tc, Monte Carlo based ^99m^Tc scatter correction was not included in the ^99m^Tc reconstruction in this method.

### Phantom study - Evaluation

Reconstructed images were evaluated based on tumor-to-normal contrast recovery, lung shunt fraction and extrahepatic activity. The contrast recovery coefficient (CRC) and lung shunt fraction (LSF) were calculated as:

1 $$CRC = \frac{{\frac{{\left\langle {{{\text{a}}_{{\text{tumor}}}}} \right\rangle - \left\langle {{{\text{a}}_{{\text{normal}}}}} \right\rangle }}{{\left\langle {{{\text{a}}_{{\text{normal}}}}} \right\rangle }}}}{{{T \mathord{\left/{\vphantom {T {N\,ratio - 1}}} \right.\kern-\nulldelimiterspace} {N\,ratio - 1}}}}$$

2 $$LSF = \frac{{{a_{lungs}}}}{{{a_{lungs}} + {a_{liver}}}}$$

Where $\left\langle a \right\rangle$ represents mean activity. Furthermore, the noise level was calculated from the liver background (i.e. normal liver tissue) consisting of N pixels as

3 $${\text{Noise}} = \frac{1}{{\left\langle {{{\text{a}}_{{\text{BG}}}}} \right\rangle }}\sqrt {\frac{{\sum\nolimits_{\text{n}}^{\text{N}} {{{({{\text{a}}_{{\text{normal}}}}\left( {\text{n}} \right) - \left\langle {{{\text{a}}_{{\text{normal}}}}} \right\rangle )}^2}} }}{{{\text{N}} - 1}}} $$

Metrics were calculated using masks for the liver background, full liver (including tumors), lungs and extrahepatic depositions, which were manually created using registered CT images and dilated by three pixels to mitigate activity spill-out. Tumor masks were defined as spheres (matching the physical volumes) centered on the center-of-mass of the highest-intensity pixels within a predefined tumor-region in the SPECT reconstruction. 3D erosion was applied to the liver background mask to exclude partial volume effects in contrast calculations, whereas the full liver mask was used for the LSF calculation.

To increase the variance estimation accuracy of the correction methods, bootstrapping was applied by resampling each synthetic projection set 25 times, resulting in 75 noise realizations per collimator [23]. Resampling was performed by redistributing all measured counts using the original projection as a probability map. Furthermore, images were visually evaluated after post-processing which was applied using a 5 mm Gaussian filter.

## Results

### Sensitivity, dead-time and quantitative accuracy

**Fig. 3:**
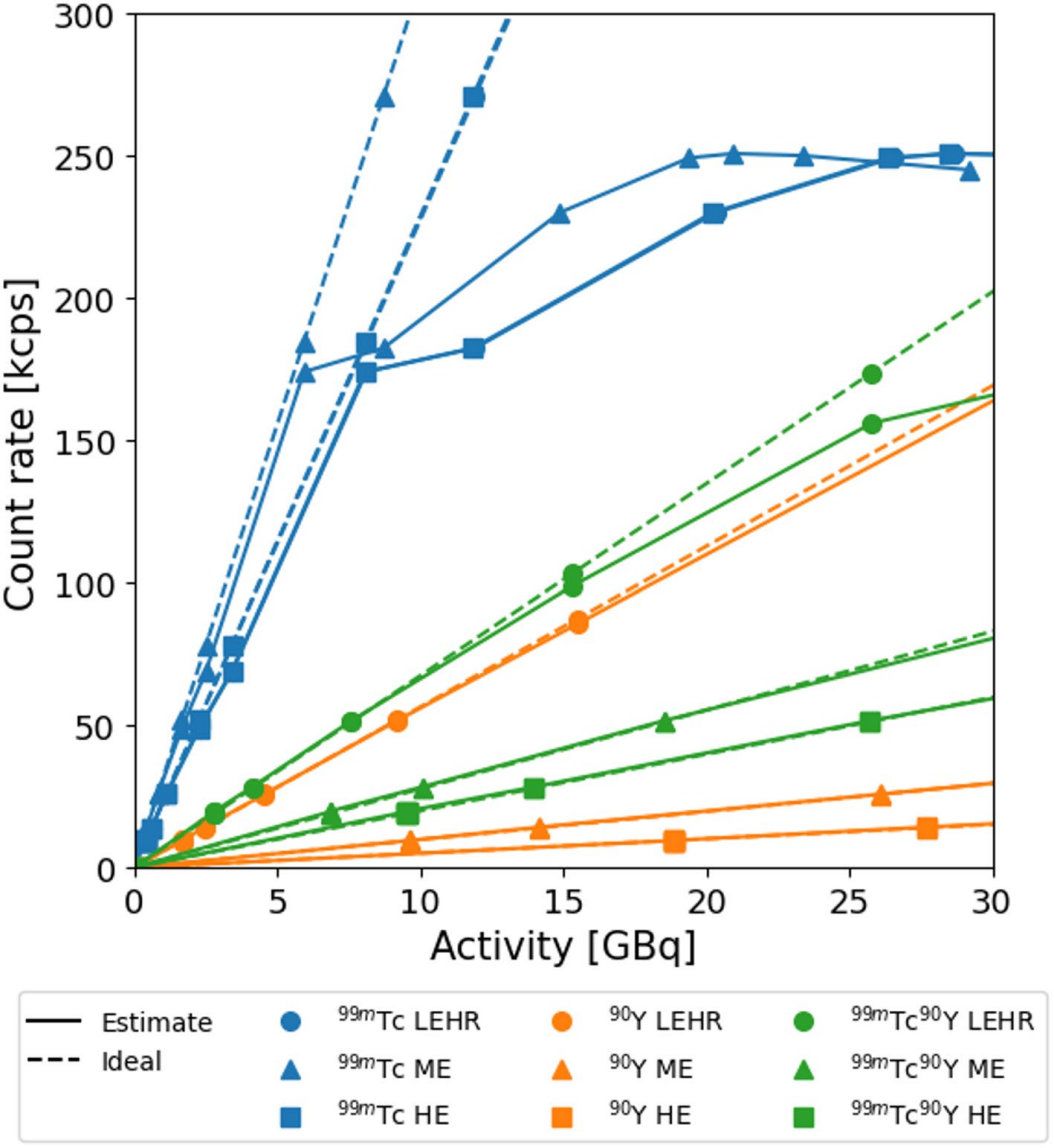
Extrinsic estimated count rate curves (solid lines) and ideal count rate slope (dashed lines) per isotope and collimator in the 140 keV window. Note: the ^99m^Tc LEHR and ^99m^Tc HE curves overlap and are therefore less distinguishable

The extrinsic sensitivities of ^99m^Tc listed in Table 2 are similar for the LEHR and HE collimator but notably higher for ME, indicating a higher collimator efficiency for ME since ^99m^Tc does not emit high energy photons. The intrinsic and extrinsic sensitivities were used to estimate the count rates at activities up to 30 GBq, as illustrated in Fig. 3. In this figure, ideal count rate trends are shown for each isotope and collimator. The estimated count rates do not substantially deviate (< 2%) from the ideal trend up to approximately 1-1.5 GBq for ^99m^Tc with any collimator, 12 GBq for ^99m^Tc+^90^Y with LEHR, 15 GBq ^90^Y with LEHR, 29 GBq for ^99m^Tc+^90^Y with ME, and > 30 GBq for the remaining combinations.

**Table 2:** Intrinsic (no collimator, full energy window) and extrinsic (per collimator, 140 kev window) sensitivities [cps/MBq]. Activity ratio between ^99m^Tc and ^90^Y was 1:20, respectively

	Intrinsic Sensitivity [cps/MBq]	Extrinsic Sensitivity [cps/MBq]		
		LEHR 140 keV	ME 140 keV	HE 140 keV
^99m^Tc	90.9e3	22.72	31.06	22.86
^90^Y	4.5e3	5.64	0.99	0.51
^99m^Tc+^90^Y	8.6e3	6.74	2.77	2.00

### Validation of synthetic dual-isotope dataset

An unpaired t-test comparing reconstructions from true dual-isotope data to those from synthetic dual-isotope data across all collimators and correction methods (MC, DEW, TEW, and no-correction) yielded *p*-values > 0.05 for CRCs, noise levels and EHD activities. LSF yielded *p*-values < 0.05 in MC-HE, TEW-LEHR, TEW-HE and no correction-HE, but *p*-values > 0.05 in all remaining cases (see Supplemental Table S1 for full results).

### Anthropomorphic Phantom study

**Fig. 4:**
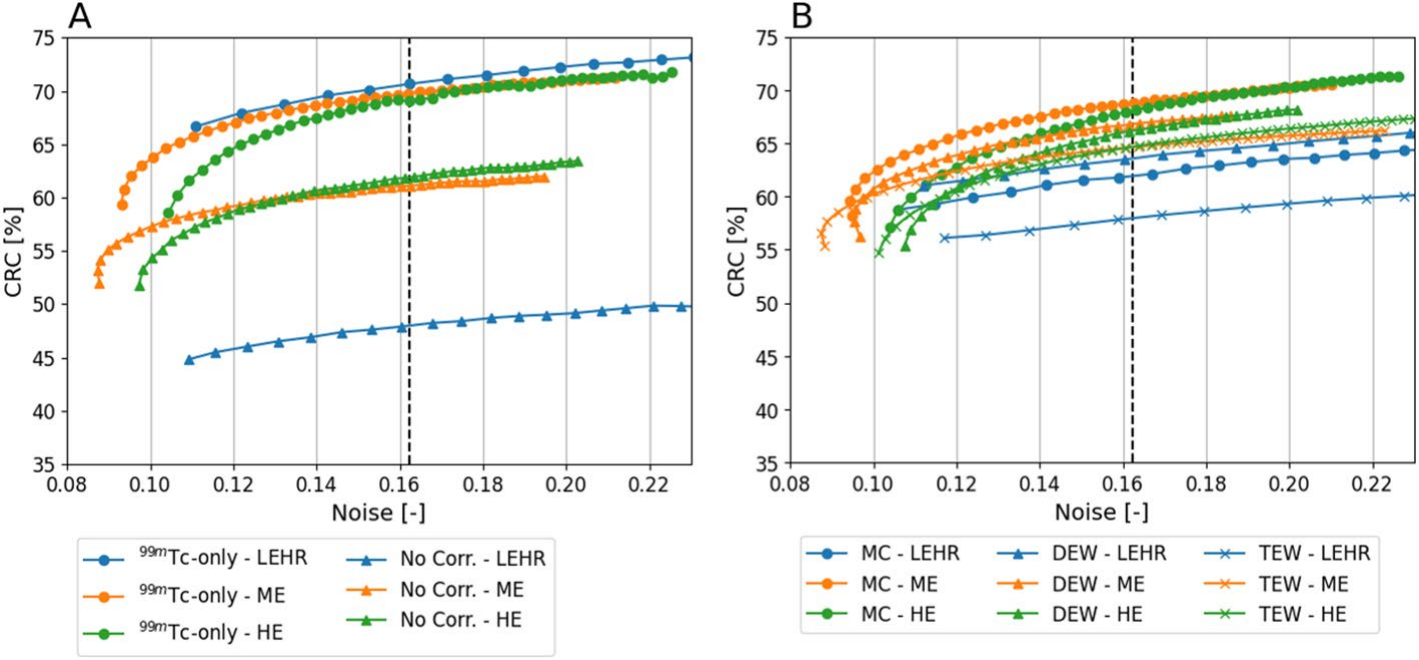
CRC (tumor 1) as function of noise level (liver background) of ^99m^Tc-only and Uncorrected dual-isotope in ^99m^Tc+^90^Y ratio of 1:20 (**A**), and MC, DEW and TEW (**B**) per collimator averaged over 3 noise realizations (i.e. without bootstrapping). Each point represents a reconstruction iteration, starting from iteration 5. The dashed line indicates the noise level (0.162) at which collimators and correction methods were compared in the phantom study

The CRC of tumor 1 and noise level of the liver background per iteration are shown in Fig. 4. All methods generally exhibit increasing noise and contrast with more iterations. Noise levels from the LEHR collimator increased substantially faster per iteration compared to ME and HE. Similar trends were observed for tumor 2.

The following results are presented at iteration numbers (per collimator and correction method) that most closely match the background noise level of the ^99m^Tc reconstruction protocol used in our hospital (10 iterations with a LEHR collimator) which was 0.162 (see Fig. 4A).

**Fig. 5:**
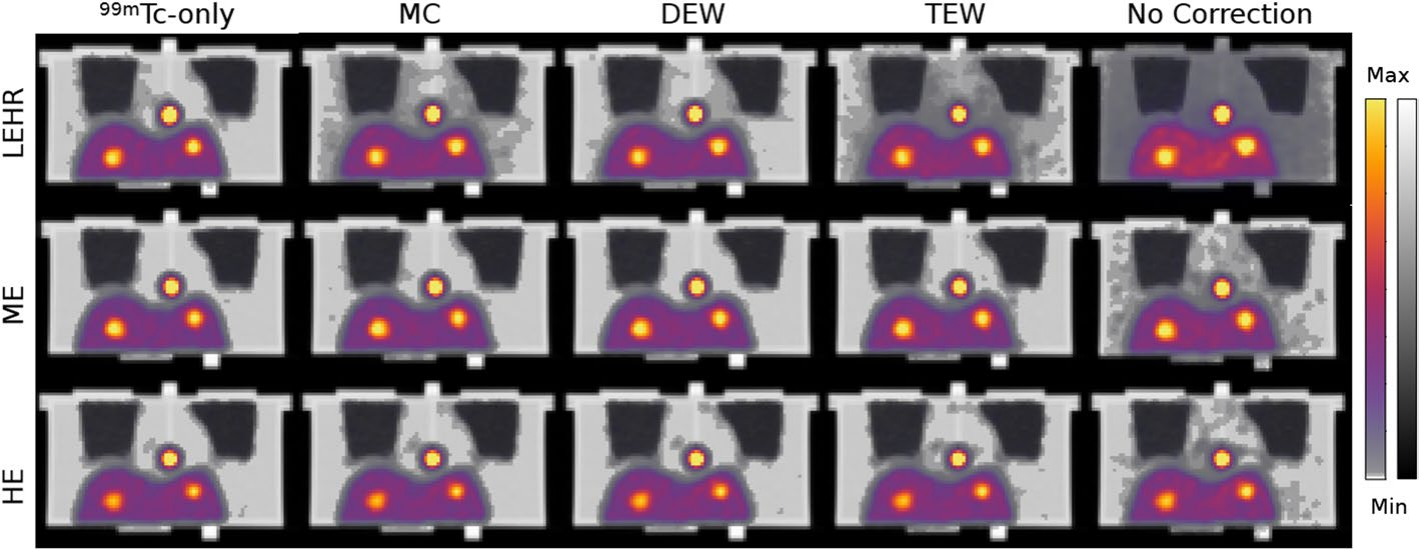
Coronal slice from the anthropomorphic phantom at calibrated amount of iterations for each reconstruction algorithm and collimator from dual-isotope data in ^99m^Tc+^90^Y ratio of 1:20. Shown are SPECT images (in color; see left color bar) merged with CT images (in gray-scale; see right color bar)

Figure 5 shows examples of coronal slices from each method and collimator combination. The LEHR collimator shows relatively high resolution images as can be seen in the ^99m^Tc-only images, but results in more contamination from ^90^Y compared to ME and HE. MC and DEW reconstruction produce the sharpest images with minimal background scatter, especially in combination with the ME collimator due to its higher effectivity compared to the HE collimator.

**Fig. 6:**
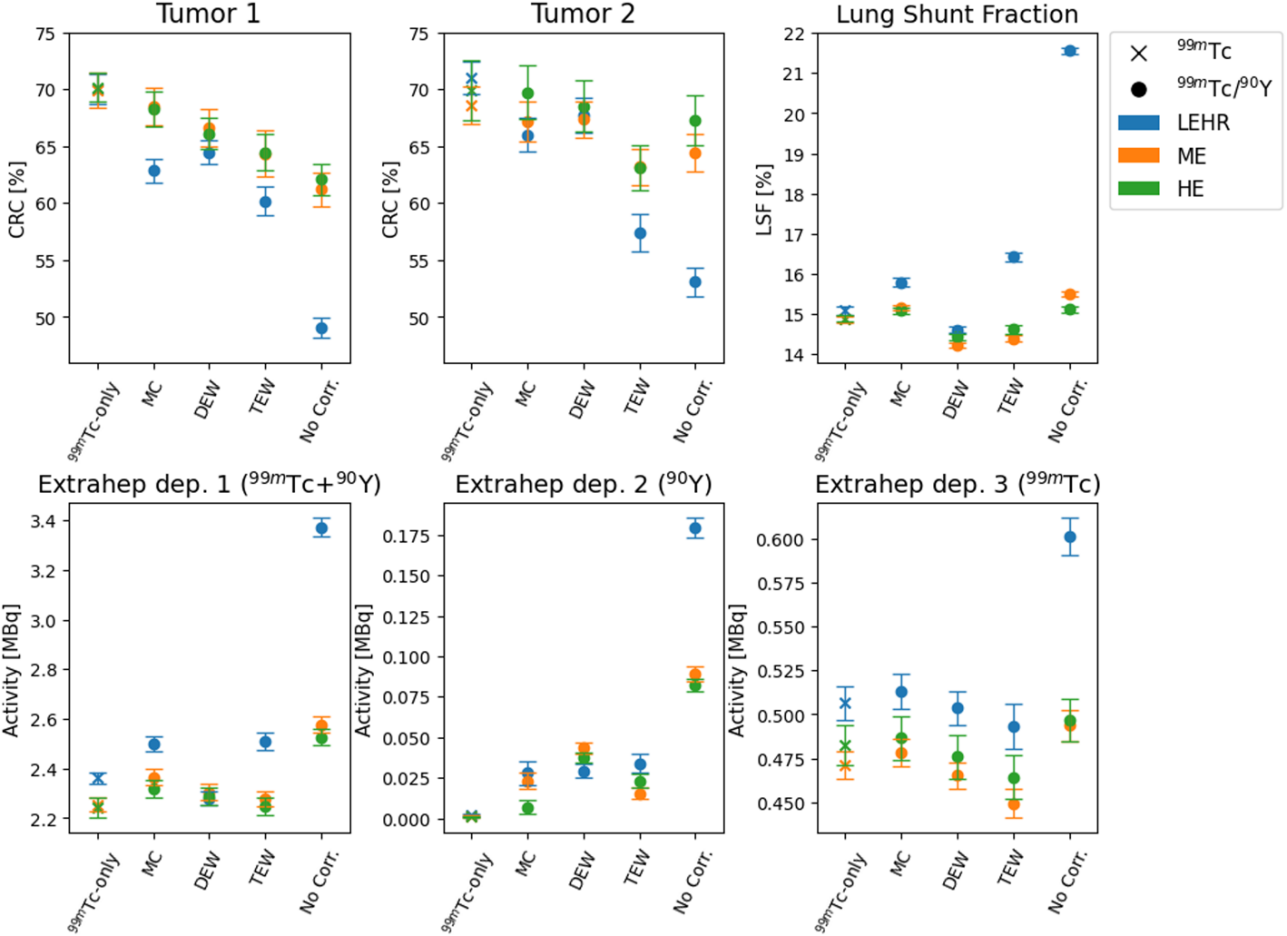
Metrics (mean value and standard deviation range over 75 noise realizations) at calibrated number of iterations (noise = 0.162) per collimator and reconstruction algorithm from ^99m^Tc-only data (cross) or dual-isotope data (dot) in ^99m^Tc+^90^Y ratio of 1:20. Metrics include CRC [%], LSF [%] and extrahepatic activities 1 (2.7 MBq), 2 (0.0 MBq) and 3 (0.7 MBq)

Figure 6 shows the mean contrast recoveries of tumor 1 and 2, the LSF, and the activities in the extrahepatic depositions. ^90^Y crosstalk correction with the MC algorithm, when used with the HE or ME collimator, produces metric values most comparable to those obtained with ^99m^Tc-only using the LEHR collimator.

**Fig. 7:**
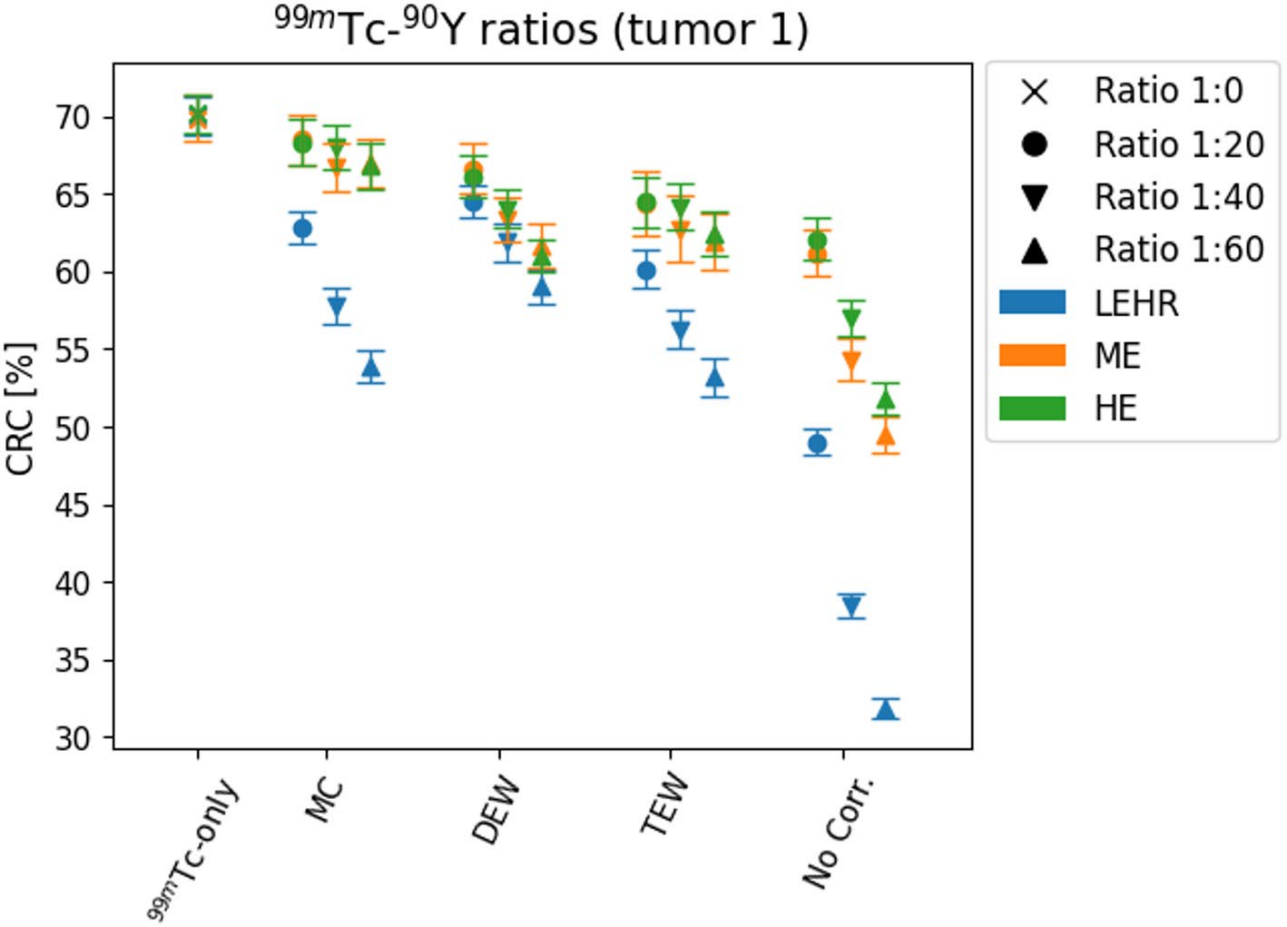
CRCs [%] of tumor 1 (mean value and standard deviation range over 75 noise realizations) at calibrated number of iterations (noise = 0.16) per collimator and reconstruction algorithm. Reconstructions are made from ^99m^Tc-only data (cross) or dual-isotope data in ^99m^Tc+^90^Y ratio of 1:20 (dot), 1:40 (inverted triangle) or 1:60 (triangle)

Figure 7 presents CRCs for two additional ^99m^Tc+^90^Y ratios (1:40 and 1:60) created by increasing ^90^Y activity in the synthetic projection data. MC and TEW correction with HE or ME collimator show less degradation at higher ^90^Y activities compared to DEW correction.

## Discussion

In this study, three methods of ^99m^Tc image reconstruction from dual-isotope data were investigated and compared. Using the clinically standard LEHR collimator for ^99m^Tc imaging as the reference, we identified the most suitable method for post-therapy ^99m^Tc imaging in the presence of ^90^Y. This situation arises when ^99m^Tc-MAA is injected during the therapy procedure. This can occur, for example, in our case of validating an innovative interventional hybrid SPECT/CT system in a clinical trial for single-session ^90^Y radioembolization. Phantom experiments revealed that the LEHR collimator was most susceptible to ^90^Y influence in ^99m^Tc image quantification. Correction for ^90^Y crosstalk with Monte Carlo simulations, or Dual Energy Window estimates as an alternative, yielded the most accurate ^99m^Tc reconstructions especially when combined with ME or HE collimators. Literature has shown that MC-based corrections can be superior to energy window-based correction, but this is mainly due to the increased noise in energy window-correction, which was not taken into account in this dosimetry-focused study [16, 24, 25].

This dual-isotope scenario may also occur in clinical scenarios where additional ^99m^Tc-MAA is administered during the ^90^Y therapeutic procedure itself, e.g. when initial MAA imaging revealed insufficient targeting of a specific liver segment [6]. Currently, such additional ^99m^Tc-MAA administrations are less common, partly due to challenges in imaging ^99m^Tc in the presence of ^90^Y. The methods proposed in this study could improve intra-procedural re-evaluation or second scout scans. This may support more adaptive treatment approaches, reduce number of interventions and lower patient burden.

A potential issue of this study is the use of synthetic dual isotope data sets generated by adding two single-isotope acquisitions to enable an equitable and in depth analysis. This was based on the assumption that single and dual isotope scans have similar linear behavior. To assert this, dead-time effects were assessed using an intrinsic-extrinsic estimation approach, similar to that of Elschot et al. (2011), in which lower activities (MBq range) can be used to estimate dead-time effects in higher activities (GBq range) [18]. Literature already showed no significant dead-time effects from ^90^Y (with ME or HE) and ^99m^Tc (with LEHR) in the clinical activity range [18, 26]. Our study showed similar results, and also extended this finding for ^90^Y using a LEHR collimator, and ^99m^Tc and dual-isotope acquisitions for all three collimators. Our results indicate that this linear behavior remains valid up to at least 29 GBq with ME and HE collimators, which already exceeds typical clinical activities. We acknowledge that patient-specific factors such as geometry were not investigated in this part of our study. Activity would likely be more spread out in complex patient geometries compared to the Eppendorf tube that was used, potentially reducing dead-time effects further. Even though not enough data was collected to determine an exact activity limit, the results show that dead-time effects causing non-linearities in the clinically relevant activity range are negligible, especially when using a ME or HE collimator.

Three collimators were considered in this study: LEHR as the standard for ^99m^Tc imaging, HE as preferred option for ^90^Y imaging, and ME serving as an intermediate alternative [9]. LEHR showed the least accurate results, presumably caused by high-energy ^90^Y photons passing through the collimator and/or insufficiently accurate ^90^Y-LEHR detector-collimator response and Monte Carlo simulations in UMCS. The HE and ME collimators yielded quantitatively more accurate reconstructions. These collimators show quantitatively negligible differences in the CRC of tumor 1, LSF and activity estimates in EHD 1 and 3, whereas the HE collimator (combined with MC correction) was superior in the CRC of tumor 2 and activity estimation of EHD 2. The ME collimator, however, outperforms the HE collimator in terms of visual contrast and sharpness, resulting in images visually more similar to the ^99m^Tc-only LEHR reconstructions. While the LEHR collimator generally provides higher spatial resolution in ^99m^Tc-only reconstructions, it is more affected by ^90^Y contamination in dual-isotope imaging, which can obscure small lesions or create false-positive uptake. ME and HE collimators, especially when combined with MC or DEW correction, reduce this contamination and background noise, improving the visibility of low-activity regions such as extrahepatic depositions. This trade-off between resolution and contamination robustness is clinically relevant, as accurate visual identification of non-target activity is critical for treatment planning and safety.

When comparing the three compensation algorithms, MC correction (in combination with HE or ME collimator) exhibits the greatest similarity to ^99m^Tc-only reconstruction across most metrics. The DEW method, a strong alternative to MC correction, substantially outperformed TEW correction and was comparable to MC in some cases (e.g., CRC of tumor 2), but was outperformed by MC in others (e.g. CRC of tumor 1). The DEW algorithm estimates ^90^Y crosstalk using a k-factor determined using the point source experiments. Arguably, the performance of this factor is coincidental and might not be robust over various patients and activities. Despite this limitation, DEW is relatively straightforward to implement in clinical workflows and, with more extensive determination of k-factors, may offer a feasible and clinically viable solution. In contrast, the MC algorithm is currently less accessible due to limited clinical availability of Monte Carlo-based reconstruction tools, but uses accurate patient-specific simulations and is therefore presumably more reliable in a clinical setting.

Our results indicate that MC and TEW correction with HE or ME collimator are the most robust options over increasing ^90^Y contamination. Additionally, utilizing the 181 keV window for ^90^Y estimation was investigated as an alternative for the 226 keV window in the MC and DEW correction methods. Keane et al. demonstrated that the 181 keV window yields ^90^Y images better suited for diagnostic purposes, but had a higher probability of ^99m^Tc contamination [10]. For this reason, we initially opted for the 226 keV window to prioritize accurate ^99m^Tc imaging. Our results showed that only DEW with HE collimator slightly benefits from using the 181 keV window, while all other combinations perform equally well with either window.

Despite certain limitations, including the use of phantom data causing limited variability in geometry and activity distributions and the reliance on Monte Carlo-based correction methods that are not yet widely available in clinical practice, this study demonstrates that the degrading effect of ^90^Y on ^99m^Tc imaging is significant and quantifiable. The findings show that this interference can be effectively mitigated by selecting an appropriate combination of collimator and correction technique, supporting the feasibility of accurate ^99m^Tc SPECT imaging in dual-isotope scenarios.

## Conclusion

In this study we investigated the feasibility and optimal method of ^99m^Tc SPECT image reconstruction from ^99m^Tc+^90^Y dual-isotope data. Physical phantom experiments demonstrated that the impact of ^90^Y crosstalk om ^99m^Tc imaging was limited when using a ME or HE collimator instead of the conventional LEHR collimator. Monte Carlo-based crosstalk correction minimizes image degradation and provides the most accurate and robust ^99m^Tc-SPECT reconstruction.

## Supplementary Information

Below is the link to the electronic supplementary material.

Supplementary Material 1

## Data Availability

The authors had full control over the data and the information submitted for publication. Data is stored at the University Medical Centre Utrecht, Utrecht, the Netherlands.

## Abbreviations

^99m^Tc: Technetium-99 m
^90^Y: Yttrium-90
CRC: Contrast recovery coefficient
CT: Computed tomography
DEW: Dual energy-window
EHD: Extrahepatic deposition
HE: High energy
LEHR: Low energy high resolution
LSF: Lung shunt fraction
MAA: Macro-aggregated albumin
MC: Monte Carlo: ME: medium energy
OSEM: Ordered-subset expectation-maximization
UMCS: Utrecht Monte Carlo system
SIRT: Selective internal radiation therapy
SPECT: Single photon emission computed tomography
TEW: Triple energy-window
T/N-ratio: Tumor to non-tumor ratio
