# Supplementary material for: Optimization of 99mTc-SPECT in the presence of 90Y for radioembolization
Source: EJNMMI Phys. 2025 Sep 25;12:84. doi: 10.1186/s40658-025-00798-5 (PMC12463788; doi:10.1186/s40658-025-00798-5)
Supplement: Supplementary file 1 — Supplementary Material 1 [file 40658_2025_798_MOESM1_ESM.docx]

| **Table S1:** p-values from unpaired t-tests comparing metrics calculated from true and synthetic dual-isotope data. Metrics include CRC from tumor 1 and tumor 2, LSF, Noise, and activity in EHD1, EHD2 and EHD3. Comparisons were performed for all correction methods (MC, DEW, TEW and no correction) and collimators (LEHR, ME, HE). P-values below 0.05 are shown in bold. | | | | | | | | | | | | |
| --- | --- | --- | --- | --- | --- | --- | --- | --- | --- | --- | --- | --- |
|  | **MC** | | | **DEW** | | | **TEW** | | | **No Correction** | | |
|  | **LEHR** | **ME** | **HE** | **LEHR** | **ME** | **HE** | **LEHR** | **ME** | **HE** | **LEHR** | **ME** | **HE** |
| **CRC tumor 1** | 0.350 | 0.821 | 0.522 | 0.222 | 0.831 | 0.720 | 0.325 | 0.634 | 0.891 | 0.360 | 0.890 | 0.839 |
| **CRC tumor 2** | 0.194 | 0.960 | 0.268 | 0.150 | 0.854 | 0.110 | 0.816 | 0.478 | 0.495 | 0.284 | 0.871 | 0.225 |
| **LSF** | 0.088 | 0.085 | **0.001** | 0.280 | 0.108 | 0.064 | **0.033** | 0.063 | **0.012** | 0.179 | 0.136 | **0.012** |
| **Noise** | 0.518 | 0.512 | 0.170 | 0.373 | 0.301 | 0.113 | 0.403 | 0.592 | 0.491 | 0.554 | 0.107 | 0.191 |
| **Activity EHD1** | 0.447 | 0.880 | 0.957 | 0.457 | 0.871 | 0.996 | 0.328 | 0.861 | 0.991 | 0.486 | 0.926 | 0.953 |
| **Activity EHD2** | 0.952 | 0.801 | 0.381 | 0.477 | 0.535 | 0.515 | 0.278 | 0.944 | 0.885 | 0.397 | 0.673 | 0.173 |
| **Activity EHD3** | 0.749 | 0.504 | 0.461 | 0.764 | 0.483 | 0.445 | 0.896 | 0.496 | 0.464 | 0.746 | 0.497 | 0.481 |
